# Elucidating the knowledge, attitude, and stigma associated with tuberculosis: a community based descriptive study in Wau and Jur River, South Sudan

**DOI:** 10.1186/s41182-025-00696-7

**Published:** 2025-02-04

**Authors:** Peter Michael Marin, Morten Tryland, Musso Munyeme, Ambrose Samuel Jubara, Enock Matovu, Peter Waiswa, Javier Sanchez Romano, Francis Mutebi, David Onafruo, Estella Kitale, Esther Sabbath, Kayla J. Buhler, Clovice Kankya

**Affiliations:** 1 Department of Public Health, University of Bahr El Ghazal (UBG), Wau, South Sudan; 2 Department of Biosecurity, Ecosystem and Veterinary Public Health, Makerere University, Kampala, Uganda; 3 Department of Forestry and Wildlife Management, Inland Norway University of Applied Sciences (INN), Evenstad, Norway; 4 Department of Arctic and Marine Biology, UiT-The Arctic University of Norway (UiT), Tromsø, Norway; 5 Department of Disease Control, University of Zambia, Lusaka, Zambia; 6 Department of Clinical Studies, University of Bahr El Ghazal (UBG), Wau, South Sudan; 7 Department of Pharmacy, Clinical and Comparative Medicine, Makerere University, Kampala, Uganda; 8 Department of Medical Biology, UiT-The Arctic University of Norway (UiT), Tromsø, Norway; 9 Department of Bimolecular Resources and Biolab Sciences, Makerere University, Kampala, Uganda

**Keywords:** Mycobacterium tuberculosis, Awareness, Driving factors, Bahr El Ghazal Region

## Abstract

**Background:**

Tuberculosis (TB) is a major public health problem in South Sudan. Inadequate knowledge, negative attitudes and perceived stigma may complicate the prevention efforts. This study describes knowledge, attitude, and stigma associated with TB among communities in Wau and Jur River, South Sudan.

**Methods:**

From March to May 2023, a cross-sectional study was conducted among 352 community members randomly selected from residential blocks. A validated structured questionnaire was used to collect the required data. Descriptive, bivariate and multivariate analyses were performed.

**Results:**

Out of 352 respondents, 51% (n = 180) were males and 49% (n = 172) were females. Majority 227 (64.5%) had poor knowledge about TB, meanwhile hearing about TB, age and level of education were associated factors. Fear of having TB was the major negative attitude (57.1%; n = 218), and most of the respondents (n = 327; 92.9%) had a perceived stigma towards TB, voicing that they disliked drinking or eating with people with TB and/or felt uncomfortable and kept their distance from people with TB.

**Conclusions:**

Communities have little knowledge, negative attitude and perceived stigma towards people with TB. Hence, tailored health messages using local languages, training of community volunteers to reach villages without accessibility and communication network are essential to improve TB prevention and control in South Sudan.

**Supplementary Information:**

The online version contains supplementary material available at 10.1186/s41182-025-00696-7.

## Introduction

Tuberculosis (TB) is a public health problem worldwide, with approximately 10.6 million cases and 1.6 million related deaths in 2022 [1–3]. The disease contributes to mortality and morbidity in South Sudan. Currently, the incidence of TB is relatively high, with 79 positive cases in every 100,000 sputum smears [4]. The disease is caused by the bacterium *Mycobacterium tuberculosis* and is transmitted through inhalation of bacilli that are coughed into the air by a patient with active TB. Tuberculosis is preventable with simple measures, such as covering the mouth and nose while coughing or sneezing and with improved hygiene and ventilation. However, the disease remains a health challenge among patients and communities in South Sudan [5].

Historically, TB has been reported among disadvantaged communities suffering from poor hygiene and sanitation, lack of awareness, co-morbidity, and inadequate health services [6, 7]. Although knowledge and attitude of communities is considered important for TB prevention efforts. For instance, behavior, motivation, or negligence of community members to follow preventative measures may lead to the spread of the disease in the community [10, 11]. However, several studies have linked poor knowledge and awareness among community members to various associated factors, including individual education level, income, or being a female participant [11–13].

Furthermore, the disease is highly associated with stigma in patients and communities worldwide [8, 9]. Stigma towards TB has been reported to have a negative impact on TB treatment and prevention [14, 15]. For example stigmatization of patients by family members, friends, co-workers and community may lead to non-adherence, further spread of the disease in the community and death [16, 17].

In South Sudan, there are limited published studies, primarily patients related. However, baseline information presented in this study provides community perspective on knowledge, attitude and perceived stigma towards TB that can help the National TB program (NTP) and relevant stakeholders in planning, monitoring and evaluating awareness activities to accelerate TB prevention and control in the country. Therefore, this study was to assess the knowledge, attitude and perceived stigma towards TB among community members in Wau and Jur River, South Sudan.

## Methods

### Study design and study area

A cross-sectional study was conducted among communities in Wau and Jur River, South Sudan, from March to May 2023. The area has a population of 127,384 people [18] and is located on the western banks of the Jur River (7°42′N, 27°59′E). The area is inhabited by several communities that mostly practice agro-pastoralism and pastoralism. The area is the second major town in the country.

### Study population

The study population was consisted of community members residing near TB treatment sites. The inclusion criteria included those who 1) were 18 years old and above, provided written informed consent, 2) were able to communicate clearly, and 3) were mentally sound. All who were less than 18 years old or declined to consent were excluded. The targeted individuals were interviewed face to face by trained research assistants.

### Sample size and sampling

The study used the formula (n = Z^2^ p (1 − p)/d^2^) to determine sample size of the participating community members.

A standard 50% prevalence of knowledge, 95% confidence interval and 5% margin of error was used. Though a sample size of 384 was determined to be sufficient, only 352 individuals were included in the final analysis as 32 respondents were excluded due to incomplete interviews.

Residential blocks near TB treatment sites were purposively sampled due to daily interaction between community members and patients with TB in these locations, while, the targeted respondents were selected randomly to participate.

### Data collection

Knowledge of the respondents was assessed using a modified WHO standard structured questionnaire based on the WHO guide to developing knowledge, attitudes and practices surveys [19].

The overall knowledge of the respondent was determined using seven main questions about the seriousness of TB infection, signs and symptoms, disease transmission, treatment and prevention. The questions were closed ended with yes/no or multiple options. Furthermore, a score of (1) was given to each correct option, and (0) was given to each incorrect or “don’t know” option. Hence, the total sum of ten (10) correct options was used to determine the level of knowledge; any participant with a score of ≤5 was classified as having poor knowledge. Attitudes and perceptions of community members were recoded using questions related to 1) their source of information about TB, 2) their feeling about having TB, 3) who to talk to about the illness, 4) where to go when found sick and 5) reasons for not going to health facility despite being sick. Perceived stigma was measured using 11 questions adopted from previous studies and TB stigma measurement guidelines [20, 21].

The level of perceived stigma was assessed using four Likert’s scale questions [20]. Overall perceived stigma was assessed via a scoring method: strongly disagree (1), disagree (2), agree (3) and strongly agree (4). The sum of the scores were calculated and respondents who scored below 23 were classified as having low perceived stigma. Those with a score above 23 were classified as having a high level of perceived stigma.

All questionnaires were pre-tested in a similar population outside the targeted study areas. The validity and internal consistency of knowledge and attitude questionnaire was 0.85 and stigma questionnaire was 0.88 using Cronbach’s alpha, where an alpha value ≥0.70 was considered satisfactory. Both instruments were designed in English and translated into Arabic language in the field verbally, by four trained and bilingual research assistants.

### Data management and statistical analysis

The data was cleaned and cross-checked for inconsistency and incompleteness. Statistical analysis was done using IBM SPSS (version 26). Participant’s characteristics were presented using frequencies and percentages. A chi-square test was used to examine the relationship between categorical variables, including age, marital status, education, and if they had heard about TB. Cronbach’s alphas tests were conducted to measure the internal reliability of the measurements, where an alpha value ≥0.7% was considered satisfactory. Logistic regression analysis was applied to assess the association between overall knowledge and socio-demographic factors. Bivariate logistic regression model was constructed using “Enter” method, for which factors with *P* ≤ 0.2 were retained. Multivariate model was constructed using “backward stepwise (Wald)” method with a *P* ≤ 0.05 for retaining a variable in the last model. Evaluation of the model was done using Hosmer–Lemeshow goodness-of fit and 95% CI.

## Results

### Socio-demographic characteristics

A total of 352 community members (Males 51%, females 49%) were interviewed. The response rate was 92%. Most of the respondents (n = 213, 60.5%) were married and were casual workers (n = 158, 44.9%). Only 12.5% (n = 44) had no formal education. More than half of the participants (n = 201, 57.1%) resided within 5 km or less of the nearest health facility (Table 1).

**Table 1:** Socio-demographic characteristics of respondents, South Sudan (n = 352)

Characteristics	Variable	Frequency	%
Sex	Male	180	51
	Female	172	49
Age	18–28 years	121	34.4
	29–38 years	186	52.8
	39–48 years	35	9.9
	Above 48 years	10	2.9
Marital Status	Single	117	33.2
	Married	213	60.5
	Widowed	15	4.3
	Divorced	7	2.0
Education	None	44	12.5
	Primary/basic	51	14.5
	Secondary	150	42.6
	Post-secondary/University	107	30.4
Occupation	Pastoralist/Farmer	88	25
	Government employee	106	30.1
	Casual employee	158	44.9
Distance from health facility	0–5 km	201	57.1
	More than 5 km	151	42.9

### Knowledge of TB among community members

Information about the knowledge of respondents is presented in Table 2. Most of the respondents had heard about TB (n = 328, 93.2%) and 91.8% (n = 323) considered TB to be a very serious disease. The main signs and symptoms of TB known to the respondents were: weight loss (n = 147, 31.3%), coughing blood (n = 140, 29.8%) and a persistent cough lasting longer than two weeks (n = 116, 24.7%). The major method of transmission of TB mentioned by community members was the cough or sneeze of a sick person (n = 231, 61.3%). Most respondents (n = 323, 91.8%) agreed that TB is treatable and identified anti-TB drugs as the method of treatment (n = 309, 90.6%). To prevent the disease, more than half of respondents suggested covering mouth and nose when coughing or sneezing (n = 201, 52.3%) and only 6.5% (n = 23) of community members considered the cost of TB treatment to be very expensive. The overall knowledge about TB was poor (n = 227, 64.5%) among the respondents (Table 2).

**Table 2:** Knowledge of respondents about TB among communities, South Sudan (n = 352)

Knowledge	Variable	Frequency	%
Heard of TB	Yes	328	93.2
	No	24	6.8
How serious is TB	Very serious	323	91.8
	Somewhat serious	20	5.6
	Not very serious	9	2.6
Common signs & symptoms of TB	Night sweats	30	6.4
	Cough that lasts longer than 2 weeks	116	24.7
	Coughing up blood	140	29.8
	Severe headache	4	0.9
	Weight loss	147	31.3
	Don’t know	33	7.0
TB mode of transmission	Through handshakes	10	2.7
	Through cough or sneeze of sick person	231	61.3
	Through sharing dishes, spoons and cups	44	11.7
	Through eating from the same plate	22	5.8
	Through touching items in public places	18	4.8
	Don’t know	52	13.8
Is TB treatable	Yes	323	91.8
	No	29	8.2
How to treat TB	Drugs specifically for TB treatment	309	90.6
	Traditional medicine or herbal	6	1.8
	Home rest without treatment	2	0.6
	Through good food	24	7.0
TB prevention	Avoiding shaking hands	16	4.2
	Covering mouth and nose when coughing or sneezing	201	52.3
	Avoiding sharing dishes, spoons and cups	60	15.6
	Washing hands after touching items in public places	78	20.3
Cost of TB treatment in the country	It’s free of charge	283	80.4
	It’s reasonably priced	29	8.2
	It’s somewhat expensive	17	4.8
	It’s very expensive	23	6.5
Overall TB knowledge	Poor	227	64.5
	Good	125	35.5

### Respondent’s attitude towards TB

More than half (n = 233, 55.2%) of respondents first heard about TB through family, friends, neighbours and colleagues. The common feelings associated with being diagnosed with TB were fear (n = 218, 57.1%), sadness or hopelessness (n = 107, 28%) and surprise (n = 23, 6%). The majority of respondents suggested that they would inform their spouse (n = 142, 36.8%), parents (n = 107, 27.7%) and/or health workers (n = 71, 18.4%) if they were diagnosed with TB, (Table 3).

**Table 3:** Attitude towards TB among respondents, South Sudan (n = 352)

Statement	Variable	Frequency	%
Source of TB information	Radio or TV	48	11.4
	Newspapers, magazines	6	1.4
	Posters	6	1.4
	Health worker, Teacher	123	29.1
	Family, friends, neighbors and colleagues	233	55.2
	Religious, community leaders	6	1.4
Feeling if found to have TB	Fear	218	57.1
	Surprise	23	6.0
	Shame	21	5.5
	Embarrassment	13	3.4
	Sadness or hopelessness	107	28.0
Person to talk to about your TB illness	Health worker	71	18.4
	Spouse	142	36.8
	Parents	107	27.7
	Child(ren)	18	4.7
	Close friends	23	6.0
	Other family members	24	6.2
	No one	01	0.3

### Perceived stigma towards TB among the community

Most of the respondents had a higher overall perceived stigma towards TB (n = 327, 92.9%). The majority of the respondents disliked eating/drinking with TB patients (n = 325, 92.3%) and felt uncomfortable staying near people with the disease (n = 318, 90.3%). Moreover, 89.2% (n = 314) of respondents suggested that they would keep their distance from people with TB and 88.6% (n = 312) suggested that they would not want their children to play with TB patients. Many community members (88.1%) had feeling of fear towards TB patients.

### Factors associated with poor knowledge of TB among community members

To determine factors associated with knowledge towards TB, a multivariate analysis was completed using the level of knowledge as the independent variable and socio-demographic characteristics as the dependent variables. After controlling for confounders, the results revealed that respondents who were above 48 years old had 86% lower odds of having poor knowledge compared to the youngest (aOR = 0.14, 95% CI 0.029–0.762). Respondents who had education background e.g. primary, secondary or university had lower odds of having poor knowledge about TB compared to those who had no education level, and those who heard about TB had 64% lower odds of having poor knowledge about TB (aOR = 0.36, 95% CI 0.146–0.895) (Table 4).

**Table 4:** Factors associated with poor knowledge of TB among communities, South Sudan (n = 352)

Variables	Level of Knowledge		Bivariate results COR(95% C I)	*P*-value	Multivariate results aOR(95% C I)	*P*-value
	Poor	Good				
Age						
18–28 years	76 (62.8%)	45 (37.2%)	1		1	
29–38 years	130 (69.9%)	56 (30.1%)	1.37 (0.847–2.230)	0.197	1.39 (0.840–2.312)	0.199
39–48 years	19 (54.3%)	16 (45.7%)	0.70 (0.329–1.504)	0.364	0.77 (0.344–1.746)	0.538
Above 48 years	02 (20.0%)	08 (80.0%)	0.14 (0.030–0.728)	0.019*	0.14 (0.029–0.762)	0.022*
Marital Status						
Single	80 (68.4%)	37 (31.6%)	1		1	
Married	137 (64.3%)	76 (35.7%)	0.83 (0.516–1.347)	0.458	-	
Widowed	06 (40.0%)	09 (60.0%)	0.30 (0.102–0.930)	0.037	-	
Divorced	04 (57.1%)	03 (42.9%)	0.61 (0.131–2.896)	0.540	-	
Education						
None	19 (43.2%)	25 (56.8%)	1		1	
Primary/basic	25 (49.0%)	26 (51.0%)	1.26 (0.562–2.846)	0.570	0.22 (0.100–0.480)	0.001*
Secondary	98 (65.3%)	52 (34.7%)	2.48 (1.250–4.918)	0.009	0.26 (0.125–0.551)	0.001*
Post-secondary/university	85 (79.4%)	22 (20.6%)	5.08 (2.381–10.856)	0.0001	0.47 (0.259–0.847)	0.012*
Heard about TB						
No	10 (41.7%)	14 (58.3%)	1		1	
Yes	217 (66.2%)	111 (33.8%)	0.36 (0.157–0.849)	0.019*	0.36 (0.146–0.895)	0.028*

*P* ≤ 0.2 were retained in bivariate and considered for multivariate analysis

^*^P ≤ 0.05 were considered statistically significant at multivariate analysis

## Discussion

Understanding the knowledge, attitude, and stigma associated with TB is critical for TB prevention and control in South Sudan. This study provided baseline information about community knowledge, attitudes, and perceived stigma towards TB. Our findings revealed significant knowledge gaps and high perceived stigma among the community. The majority had poor knowledge about TB signs and symptoms, transmission, treatment and prevention. Some respondents suggested that handshakes, sharing of personal items, touching things or eating from the same plate with a sick person are sources of TB transmission. This deficiency in knowledge is consistent with other studies conducted in Myanmar and Tanzania, which reported poor knowledge about TB in affected communities [12, 17]. However, in South Africa, nearly three quarters of community members appear to have adequate knowledge about TB [22]. This discrepancy between countries might be due to a higher literacy rate and better access to information contrary to the situation among the community members in South Sudan.

Moreover, health workers rarely provide TB information to this community, which also contributes to a lack of knowledge about the disease. Although most community members in this study were aware of the treatment options including modern TB drugs, however, a few suggested the use of traditional herbs, getting enough rest at home, or good nutrition. These findings are in agreement with studies conducted in Gambia, Ethiopia, Malawi and Pakistan respectively, that showed negative attitudes and perceived stigma towards persons with TB [7, 9, 11, 13, 15]. Thus, increasing accessibility to information, such as enhancing efforts from health workers to disseminate correct information about TB to communities, would likely reduce the knowledge gaps identified in this study.

Furthermore, it is understood that: the age group and the education are important in shaping the knowledge and the experience of the individuals about the diseases including TB. For instance, the community members who heard about TB may develop awareness and proper understanding of TB. Our findings were similar to previous studies conducted in Myanmar, Gambia [11, 17].

Generally, community members reported negative attitudes towards TB, including feelings of fear, sadness, hopelessness and surprise. These findings concurs with earlier studies conducted in Gambia (2020) and Ethiopia (2019).

In this study, more than half of the community members heard about TB from people surrounding them such as family, friends, neighbors and colleagues, etc. Similar findings were described in studies from Ethiopia and Gambia [11, 23]. Sharing information through informal means (e.g. family, friends, and neighbours) is rooted in the culture and values of openness and togetherness among South Sudanese communities. Secondly, health workers and teachers are community gatekeepers that provide trustworthy information and guidance. Despite technological advancements in other regions of the world, the radio is still considered to be the most influential media source for information sharing in Wau and Jur River. This emphasizes the importance of understanding cultural practices and accessibility of technology in each region to effectively disseminate efforts to increase knowledge about TB.

The stigma associated with TB is a hindrance to early detection and can lead to late referral and treatment among community members [15, 16]. In this study, stigmatization and discrimination against people with TB in the community was high, which can lead to increased transmission [24]. Our findings were in agreement with studies conducted in India and Cambodia, which also revealed stigmatizing and discriminating attitudes towards TB patients [16, 25]. We understand that the major reason for stigma associated with TB among communities in Wau and Jur River was misconception and lack of accurate information about TB in the community. However, cultural beliefs such as considering cough not infectious and the use of traditional medicine and witchcraft have also contributed to the spread of the disease in the community. Therefore, health workers and national TB program (NTP) staff should urgently implement or strengthen social mobilization and awareness campaigns at the community level to raise awareness for TB prevention and control.

This study has few limitations; a lack of qualitative approach could have helped in validation of quantitative information reported here. Also, there is a possible bias due to recall and self-reporting responses. Furthermore, the current findings may not be generalized to wider communities because of purposive sampling of the study population.

## Conclusions

Overall, the respondents had poor knowledge, and negative attitude. The perceived stigma against persons with TB was high. However, increased age, education status, and awareness (hearing about TB) were associated with good knowledge about TB. There were also negative attitudes towards persons with TB among the community members. Thus, tailored health messages using local languages, training of community volunteers to reach villages without accessibility and communication network are recommended to accelerate TB prevention and control in South Sudan.

## Supplementary Information

Additional file 1.

Additional file 2.

## Data Availability

No datasets were generated or analysed during the current study.

## Abbreviations

TB: Tuberculosis
NTP: National Tuberculosis Program
CI: Confidence Interval
WHO: World Health Organization
COR: Crude Odd Ratio
aOR: Adjusted Odd Ratio
ACSM: Advocacy, Communication and Social Mobilization
