# Supplementary material for: Elucidating the knowledge, attitude, and stigma associated with tuberculosis: a community based descriptive study in Wau and Jur River, South Sudan
Source: Trop Med Health. 2025 Feb 4;53:15. doi: 10.1186/s41182-025-00696-7 (PMC11796033; doi:10.1186/s41182-025-00696-7)
Supplement: Supplementary file 1 — Additional file 1. [file 41182_2025_696_MOESM1_ESM.pdf]

INFORMED CONSENT FORM

(For adults research participants aged 18 years and above)

TITLE OF THE STUDY: Knowledge, attitudes and stigma associated with tuberculosis: a cross- sectional study among communities in Wau Municipality, South Sudan

INTRODUCTION: My name is **Peter Michael Marin** a researcher from **Makerere University** (Uganda) **and University of Bahr el Ghazal, college of Public and Environmental Health** (South Sudan), I would like to learn about knoweldge, attitudes and perceived stigma among community members in Wau, WBGS-South Sudan.

PURPOSE OF THE STUDY: This study will investigate the respondents knowledge, attitudes and stigma associated with TB in Wau, South Sudan. We would like to understand the level of knowledge, attitudes and perceived stigma associated with TB among community members in the area and this will help in developning prevention and awareness interventions and will provide guidance to NTP . Therefore, your participation is important and **voluntary** to help us to acheive the purpose of this study. The information obtained will be used solely for educational and research purposes.

STUDY PROCEDURES: If you agree to participate in this study, we will ask you few questions through the questionnaire, the questionnaire is expected to take 10-15 mintues only. **STUDY DURATION:** This study data collection will is only once and no more interviews again.

STUDY PARTICIPANTS AND STUDY LOCATION

This study is targeting community members at selected residential blocks, who are 18 years and above, mentally sound and able to understand the study requirements and consent to participate. The sample size for this study is 352 community members, the participants will be interviewed once.

RISKS/DISCOMFORTS:

There is no known risks associated with this study. However, if you may feel not comfortable for any reasons you can feel free not to answer/or to withdraw from the participation.

BENEFITS OF THE RESEARCH STUDY:

The findings of this research will help healthcare workers and National TB program to improve TB treatment and prevention to you, State, country and the world.

COST:

You will not pay any money for your participation in this study.

COMPENSATION FOR PARTICIPATION IN THE STUDY:

No any compensation for your time participating in this study and it's solely voluntary.

QUESTIONS ABOUT THE STUDY:

In case you have any other questions related to the study or to your participation you may contact Mr **Peter Michael Marin**, the Principal Investigator of the study or may reach him at University of Bahr el Ghazal, College of Public and Environmental Health, Wau or contact +211915848061.

QUESTIONS ABOUT PARTICIPANTS RIGHTS:

Should you have questions related to your rights as a research participant that have not been answered by the Principal Investigator or if you wish to report any concerns about the study you can contact Director General, Ministry of Health, Western Bahr El Ghazal State.

STATEMENT OF INFORMED CONSENT:

You participation in this study is voluntary and you may join on your free will and you have a right to withdraw at any time without penalty. Do you agree to take part in this study?

RESPONDENT ANSWER?

Yes ………………………..

No ………………………...

STATEMENT OF CONSENT

I understand the procedures described above. My questions have been answered to my satisfaction and I agree voluntary to take part in this study and I reserve the right to withdraw at any time if I will.

Name of research participant……………………………………………Age………………..

Signature/thumbprint

Date ………………………
