# Supplementary material for: Elucidating the knowledge, attitude, and stigma associated with tuberculosis: a community based descriptive study in Wau and Jur River, South Sudan
Source: Trop Med Health. 2025 Feb 4;53:15. doi: 10.1186/s41182-025-00696-7 (PMC11796033; doi:10.1186/s41182-025-00696-7)
Supplement: Supplementary file 2 — Additional file 2. [file 41182_2025_696_MOESM2_ESM.pdf]

Questionnaire about the Knowledge, attitudes and perceived stigma among communities in Wau, Western Bahr el Ghazal State - South Sudan.

Questionnaire Number (   ) **Date of Interview ………………………………**

**Socio – demographic Information:**		
**S/No**	**Questions**	**Location (GPS): ………………………………………………………**
SD/A1	Age	1. 18-28 years ☐<br>2. 28-38 years ☐<br>3. 38-48 years ☐<br>4. above 48 years ☐
SD/A2	Sex	1. Male ☐<br>2. Female ☐
SD/A3	Marital status	1. Single ☐<br>2. Married ☐<br>3. Widowed ☐<br>4. Divorced ☐
SD/A4	Education level	1. None ☐<br>2. Primary/basic ☐<br>3. Secondary ☐<br>4. Post-secondary/University ☐
SD/A5	Occupation	1. Pastoralist ☐<br>2. Farmer ☐<br>3. Government employee ☐<br>4. Private employee ☐
SD/A6	Ethnicity	1. ☐<br>2. ☐
SD/A7	How far do you live from the nearest health facility or hospital?	1. 0- 5 Kilometers ☐<br>2. More than 5 Kilometers ☐
**TB knowledge and awareness**		
KA/B1	Have you ever heard about TB?	1. Yes ☐<br>2. No ☐
KA/B2	Do you know that TB can affect both human and animal?	1. Yes ☐<br>2. No ☐
KA/B3	Where did you first learn about tuberculosis TB?	1. Radio /TV ☐<br>2. Newspapers / magazines ☐<br>3. Posters ☐<br>4.Health worker/Teacher ☐<br>5. Family, friends, neighbours and colleagues ☐ ☐<br>7. Religious, community leaders ☐<br>8. Others ☐
KA/B4	In your opinion, how serious is TB? (Check one)	1. Very serious ☐<br>2. Somewhat serious ☐

		3. Not very serious
KA/B5	What are the signs and symptoms of TB? (Please check all that are mentioned)	1.Night sweats<br>2. Cough that lasts longer than 2 weeks<br>3. Coughing up blood<br>4. Server headache<br>5. Weight loss<br>6. Don't know
KA/B6	How can a person get TB from another person? (Please check all that are mentioned)	1. Through handshakes<br>2. Through cough or sneeze of sick person<br>3. Through sharing dishes, spoons and cups<br>4. Through eating from the same plate<br>5. Through touching items in public places<br>6. Don't know
KA/B7	How can a person prevent getting TB? (Please check all that are mentioned)	1. Avoid shaking hands<br>2. Covering mouth and nose when coughing or sneezing<br>3. Avoiding sharing dishes, spoons and cups<br>4. Washing hands after touching items in public places<br>5. Through good nutrition/food<br>7. Don't know
**TB attitudes and care-seeking behavior**		
KA/B8	Where do you usually go if you are sick? (Check all that are mentioned)	1. Private clinic<br>2. Government facility/hospital<br>3. Traditional healer<br>4. Others
KA/B9	Is TB treatable?	1. Yes<br>2. No
KA/B10	If yes, how can someone with TB be treated? (Please check all that are mentioned)	1. Drugs specifically for TB<br>2.Traditional medicine (herbal-remedies)<br>3. Home rest without medicine<br>4. Through good nutrition/food<br>5. Don't know
KA/11	How would you feel if you were found to have TB?	1. Fear<br>2. Surprise<br>3. Shame<br>4. Embarrassment<br>5. Sadness or hopelessness<br>6. Others
KA/12	Who would you talk to about your illness if you had TB?	1. Health worker<br>2. Spouse

		3. Parents<br>4. Child (ren)<br>5. Close friend<br>6. Other family members<br>7. No one<br>8. Others
KA/B13	What would you do if you had TB symptoms?	1. Go to health facility<br>2. Go to Pharmacy<br>3. Go to traditional healer<br>4. Purse other self-treatment options<br>5. Others
KA/B14	If you would not go to the health facility, what is the reason? (Please check all that apply)	1. Not sure where to go<br>2. Cost of TB treatment<br>3. Difficulties with transportation & distance to clinic<br>4. Don't trust health workers<br>5. Don't like attitude of health workers<br>6. Cannot leave work due to overlapping of time<br>7. Don't want to find out that something is really wrong<br>8. Others (Explain)
KA/B15	How expensive do you think TB diagnosis and treatment is in this community?	1. It is free of charge<br>2. It is reasonably priced<br>3. It is somewhat/moderately expensive<br>4. It is very expensive
KA/B16	Do you know somebody/person who have/had TB?	1. Yes<br>2. No
KA/B17	What are the source of information that you think can most effectively reach people like you with information on TB (Please chose the 3 most effective sources)?	1. Radio /TV<br>2. Newspapers / magazines<br>3. Posters<br>4.Health worker/Teachers<br>5. Family, friends, neighbours and colleagues<br>6. Religious, community leaders<br>7. Others

KA/B18**-** Do you think you can get TB? (Please ask a respondent to explain his/her answers)

Yes (because)………

No (because) ………..

**Community perspective towards TB related stigma**<br>**S/C**. Please ask the participants place (×) or tick (√) a number which best represents your answer.					
S/No	Questions	Strongly disagree (0)	Disagree (1)	Agree (2)	Strongly Agree (3)
S/C1	Some people may not want to eat or drink with friends who have TB.				
S/C2	Some people feel uncomfortable about being near those with TB.				
S/C3	If a person has TB, some community members will behave differently towards that person for the rest of his/her life.				
S/C4	Some people do not want those with TB playing with their children.				
S/C5	Some people keep their distance from people from people with TB.				
S/C6	Some people think that those with TB are disgusting.				
S/C7	Some people do not want to talk to others with TB.				
S/C8	Some people are afraid of those with TB.				
S/C9	Some people try not to touch others with TB.				
S/C10	Some people may not want to eat or drink with relatives who have TB.				
S/C11	Some people prefer not to have those with TB living in their community.				

Thank you for your time!
